# Mechanical thrombectomy for symptomatic stent thrombosis after carotid artery stenting

**DOI:** 10.3389/fneur.2023.1277366

**Published:** 2023-11-08

**Authors:** You-Min Fan, Han-Yang Liu, Yu-Yang Xue

**Affiliations:** ^1^Department of Neurology, Xuzhou Municipal First People’s Hospital, Xuzhou, China; ^2^Department of Interventional Radiology, Xuzhou Central Hospital, Xuzhou, China; ^3^Department of Interventional Radiology, Xuzhou Municipal First People’s Hospital, Xuzhou, China

**Keywords:** Carotid artery stenosis, carotid stent thrombosis, mechanical thrombectomy, ischemic stroke, carotid artery stenting

## Abstract

**Background:**

As there is still no consensus on the treatment of carotid stent thrombosis (CST), we would like to describe our experience with the revascularization of CST by mechanical thrombectomy.

**Methods:**

We retrospectively studied patients who underwent mechanical thrombectomy after CST at Xuzhou Municipal First People’s Hospital and Xuzhou Central Hospital between January 2020 and November 2022. The results of the procedures, complications, and clinical and imaging follow-up were recorded.

**Results:**

A total of six patients were included in this study. The stenosis grade before stent implantation was ≥85% in all patients, and the stenosis length ranged from 7 to 20 mm. Patients experienced CST within 6 days to 45 months after carotid artery stenting (CAS); the median admission on the National Institutes of Health Stroke Scale (NIHSS) at CST was 12 (range 8–25). Mechanical thrombectomy was successfully performed in all patients. There was no periprocedural death, and the modified Rankin Scale (mRS) at the 3-month follow-up was 0–2. All patients showed recovery from their neurological deficits.

**Conclusion:**

The treatment of symptomatic CST with mechanical thrombectomy resulted in satisfactory clinical outcomes. This regimen could be effective and safe, and future prospective and randomized studies are warranted.

## Introduction

Ischemic stroke has become a major public health problem worldwide, with high mortality and recurrent incidence (1), and carotid stenosis is an important factor in recurrent ischemic stroke (2). Carotid endarterectomy (CEA) is regarded as the standard treatment regimen, and best medical treatments (BMTs) such as antiplatelet and antihyperlipidemic therapies have also been used to protect patients from stroke. In addition, carotid artery stenting (CAS) is now considered an alternative regimen for the revascularization of carotid stenosis with high efficacy and safety (3).

Previous studies have reported the complications induced by CAS, including postoperative carotid in-stent restenosis (ISR), carotid stent thrombosis (CST), hyperperfusion encephalopathy (HPE), and intracranial hemorrhage (4), of which CST is a relatively rare event (0.04–2.0%) but potentially fatal due to neurological deficits; it is categorized as either early (≤ 1 month), late (> 1 month), or very late (> 12 months) (4, 5). However, there is no definitive treatment strategy for CST, and only a few case reports have ever addressed the treatment regimen for revascularization after CST, such as the administration of argatroban, thrombolytic therapy, thrombus recovery, and stent removal (5, 6). There is an urgent need to find an effective and safe approach to preventing the catastrophic neurological consequences of CST.

In this study, we retrospectively present our experience in managing carotid stent thrombosis following carotid stenting and conclude that mechanical thrombectomy may be an alternative option for patients with symptomatic stent thrombosis after carotid stenting.

## Materials and Methods

### Patients

This study was performed according to the guidelines of the Xuzhou Municipal First People’s Hospital and the Xuzhou Central Hospital Ethics Committee. Only patients who agreed with and signed the informed consent form were included in our study. We retrospectively analyzed patients who experienced severe symptomatic stent thrombosis (National Institutes of Health Stroke Scale, NIHSS ≥6) at Xuzhou Municipal First People’s Hospital and Xuzhou Central Hospital from January 2020 to November 2022. In all the included patients, CST was treated with mechanical thrombectomy. Patients who received other treatment regimens, including carotid endarterectomy, alteplase, and/or urokinase thrombolysis were excluded. Moreover, patients with severe cardiac, hepatic, and/or renal impairments were also excluded from the study.

### Mechanical thrombectomy

Mechanical thrombectomy used for treating the patients was performed by professional interventional neuroradiologists as follows: During the study period, endovascular techniques remained largely unchanged and predominantly involved an antegrade approach for treating in-stent carotid artery occlusions with aspiration and angioplasty, while treating the distal occlusion when necessary. In brief, all patients underwent general anesthesia for the procedure. Carotid stent thrombosis was confirmed by diagnostic angiography. After the insertion of an 8-Fr guide catheter into the distal common carotid artery, occlusion was evaluated using a microcatheter and a 0.014-inch guide wire. The location and extent of the thrombus were assessed by microcatheter angiography. If there was enough space at the distal end of the thrombus, the carotid protection device (CPD) was positioned in the internal carotid artery (ICA). The reperfusion catheter was then advanced into the stent to aspirate the thrombus, and, if necessary, post-dilation of the stenosis was performed using an appropriate monorail angioplasty balloon. The distal occlusion was then managed with a stent retriever, aspiration, or a combination of both techniques.

### Antiplatelet therapy

Before stent implantation, all the patients were recommended to take antiplatelet drugs such as aspirin 100 mg/day and clopidogrel 75 mg/day for 4 days, followed by continuous dual antiplatelet therapy and then aspirin alone based on clinical and imaging assessment.

### Neurological and imaging evaluation

Neurological deficits were assessed by professional neurologists at baseline, during the procedure, at discharge, and at 3-month follow-up according to the National Institutes of Health Stroke Scale (NIHSS) and the modified Rankin Scale (mRS). Radiological data from brain magnetic resonance imaging (MRI), computed tomography angiography (CTA), and computed tomography perfusion (CTP) were also collected, including baseline, periprocedural, and outpatient neuroimaging.

## Results

### Recruitment procedure

**Figure 1:**
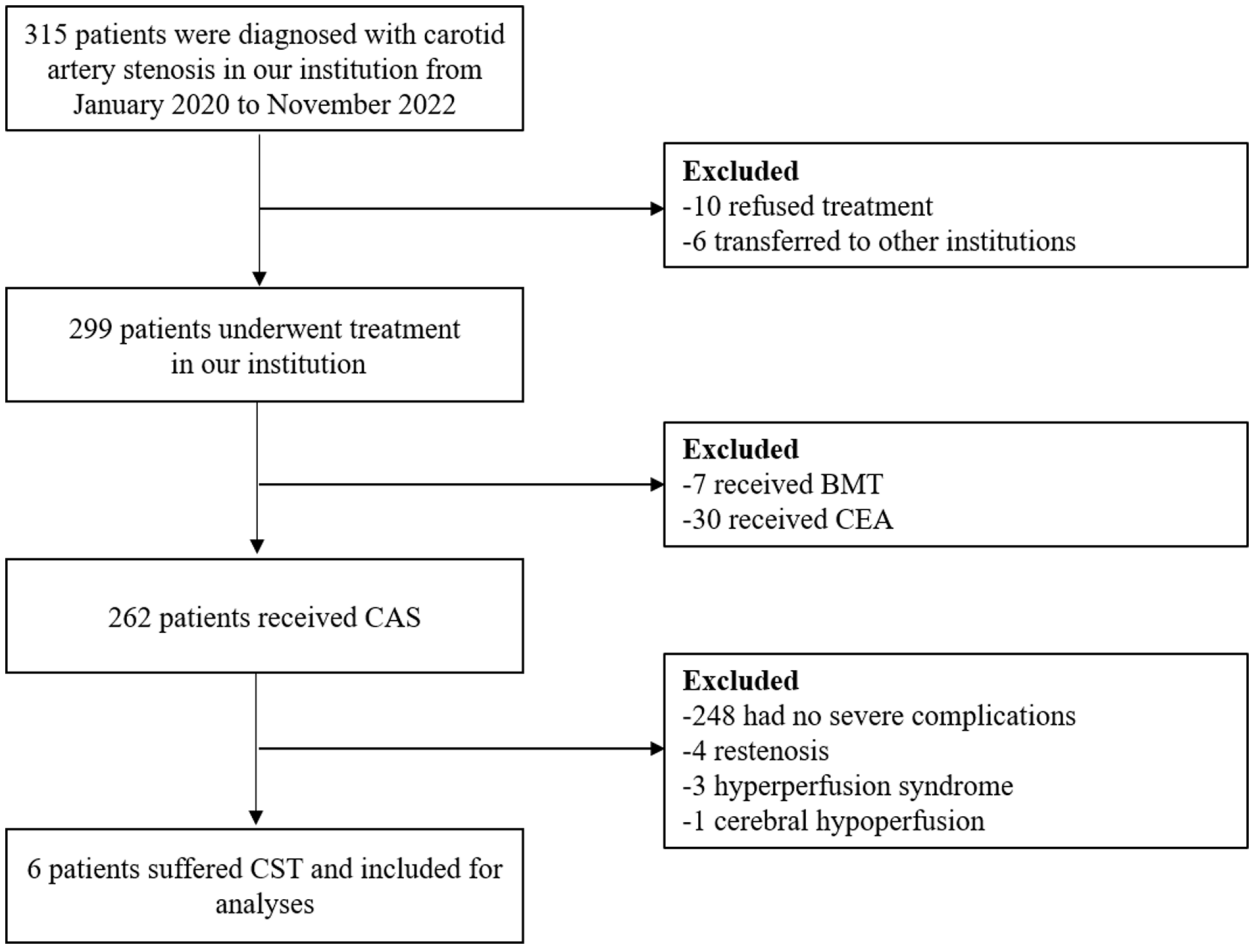
Flowchart of the recruitment process. BMT, best medical treatment; CEA, carotid endarterectomy; CAS, carotid artery stenting; CST, carotid stent thrombosis.

The enrollment process for the study is shown in Figure 1. From January 2020 to November 2022, a total of 315 patients were diagnosed with carotid artery stenosis in our institution, of which 262 patients underwent CAS, and 30 and 7 patients were treated with CEA and BMT, respectively. After revascularization by stenting, 248 (94.7%) patients had no severe complications, 4 CAS patients developed restenosis, 3 patients suffered from hyperperfusion syndrome, and 6 (2.3%) patients experienced stenting thrombosis and received mechanical stent thrombectomy.

### Patients’ characteristics

Patients’ baseline characteristics before CAS are summarized in Table 1. The median age of the participants was 69.5 years (range 55–80 years), and six patients were men. Patients had different risk factors (100% for smoking and hyperhomocysteinemia, 66.7% for hypertension and hyperlipidemia, and 33.3% for diabetes mellitus). The left carotid artery was affected by 100%. The carotid artery stenosis grade was ≥85% (range 85–95%) in all patients, and the stenosis length ranged from 7 to 20 mm. All patients presented with neurological deficits, such as limb weakness, slurred speech, and dizziness, and there was no significant improvement after adequate medical therapy. A total of four patients underwent CAS with the XACT carotid stent (Abbott Vascular, Santa Clara, California, United States), and two patients were treated with the Acculink stent system (Abbott Vascular, Cedex, France). After the procedure, the CTA results showed that the degree of stenosis was reduced to 30–40%. All patients were recommended to take antiplatelet drugs such as aspirin and clopidogrel after CAS.

**Table 1:** Baseline characteristics of enrolled patients.

Patients’ No.	Age (years)	Gender	Smoking	Alcohol drinking	Hyper- tension	Diabetes	Hyper- lipidemia	Hyper- homocysteinemia	Lension side	Stenosis Grade (%)	Stenosis length (mm)	Initial symptoms	Stent type ^a^	CPD	Postprocedural antiplatelet	Stenosis grade after CAS (%)
1	78	Male	Yes	Yes	Yes	No	Yes	Yes	Left	90	15	Right limb weakness	Acculink Stent System 7–10*40 mm	Yes	ASA + CLO	30
2	70	Male	Yes	No	Yes	Yes	Yes	Yes	Left	90	19	Slurred speech	XACT carotid stent 7–9*40 mm	Yes	ASA + CLO	35
3	69	Male	Yes	No	Yes	Yes	No	Yes	Left	95	20	Slurred speech, right limb weakness	XACT carotid stent 7–9*40 mm	Yes	ASA + CLO	40
4	63	Male	Yes	No	No	No	Yes	Yes	Left	90	8	Right limb numbness	Acculink Stent System 7–10*40 mm	Yes	ASA + CLO	40
5	73	Male	Yes	Yes	No	No	Yes	Yes	Left	85	7	Slurred speech	XACT carotid stent 7–9*40 mm	Yes	ASA + CLO	35
6	67	Male	Yes	Yes	Yes	No	No	Yes	Left	95	10	Dizziness, slurred speech	XACT carotid stent 7–9*40 mm	Yes	ASA + CLO	40

CPD, carotid protection device; ASA, aspirin; CLO, clopidogrel; CAS, carotid artery stenting. ^a^Stent types: XACT carotid stent (Boston Scientific, Natick, MA, United States); Acculink Stent System (Abbott Vascular, Cedex, France).

### Clinical follow-up results

As shown in Table 2, two patients developed symptomatic deficits approximately 1 week after CAS, and another four patients were diagnosed with CST 3 months later, with five patients failing to take adequate antiplatelet drugs. The median NIHSS after CST was 12 (range 8–25). Mechanical thrombectomy was successfully performed in all six patients with symptomatic stent thrombosis, and one patient had distal intracranial occlusion at proximal middle cerebral artery 1 (M1). At the time of discharge, the median NIHSS score was 2 (range 0–6). In the 90 days after the mechanical thrombectomy, there were no deaths and no ischemic or hemorrhagic events. The mRS at the 3-month follow-up was 0 in five patients, with only one patient suffering residual aphasia with an mRS of 2.

**Table 2:** Summary of peri-CST treatment and clinical follow-up.

Patients’ No.	Time after CAS	Time of antiplatelet discontinue	Cause of CST	Neurological deficits after CST	NIHSS after CST	Recanalization	Outcome	NIHSS at discharge	mRS at 3 months
**1**	45 months	1 month	Antiplatelet discontinue	Coma	25	Yes	Clinical status improvement	0	0
**2**	6 days	2 days	Antiplatelet discontinue	Slurred speech, right limb weakness	8	Yes	Clinical status improvement	2	0
**3**	8 days	1 day	Antiplatelet discontinue	Hypersomnia, aphasia, right hemiplegia	20	Yes	Clinical status improvement	4	0
**4**	20 months	–	Not determinded	Aphasia, right limb weakness	12	Yes	Remained residual aphasia	6	2
**5**	12 months	7 days	Antiplatelet discontinue	Slurred speech, right limb weakness	10	Yes	Clinical status improvement	0	0
**6**	3 months	3 days	Antiplatelet discontinue	Slurred speech, right limb weakness	12	Yes	Clinical status improvement	2	0

CST, carotid stent thrombosis; CAS, carotid artery stenting; NIHSS, National Institutes of Health Stroke Scale; mRS, modified Rankin Scale.

### Illustrative case

**Figure 2:**
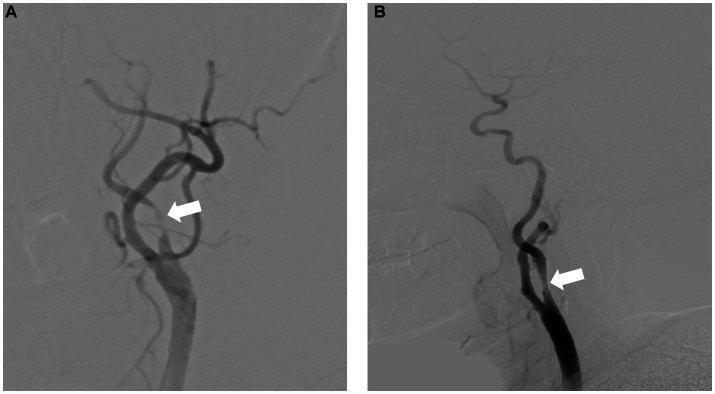
Severe carotid artery stenosis before **(A)** and after **(B)** successful carotid artery stenting.

**Figure 3:**
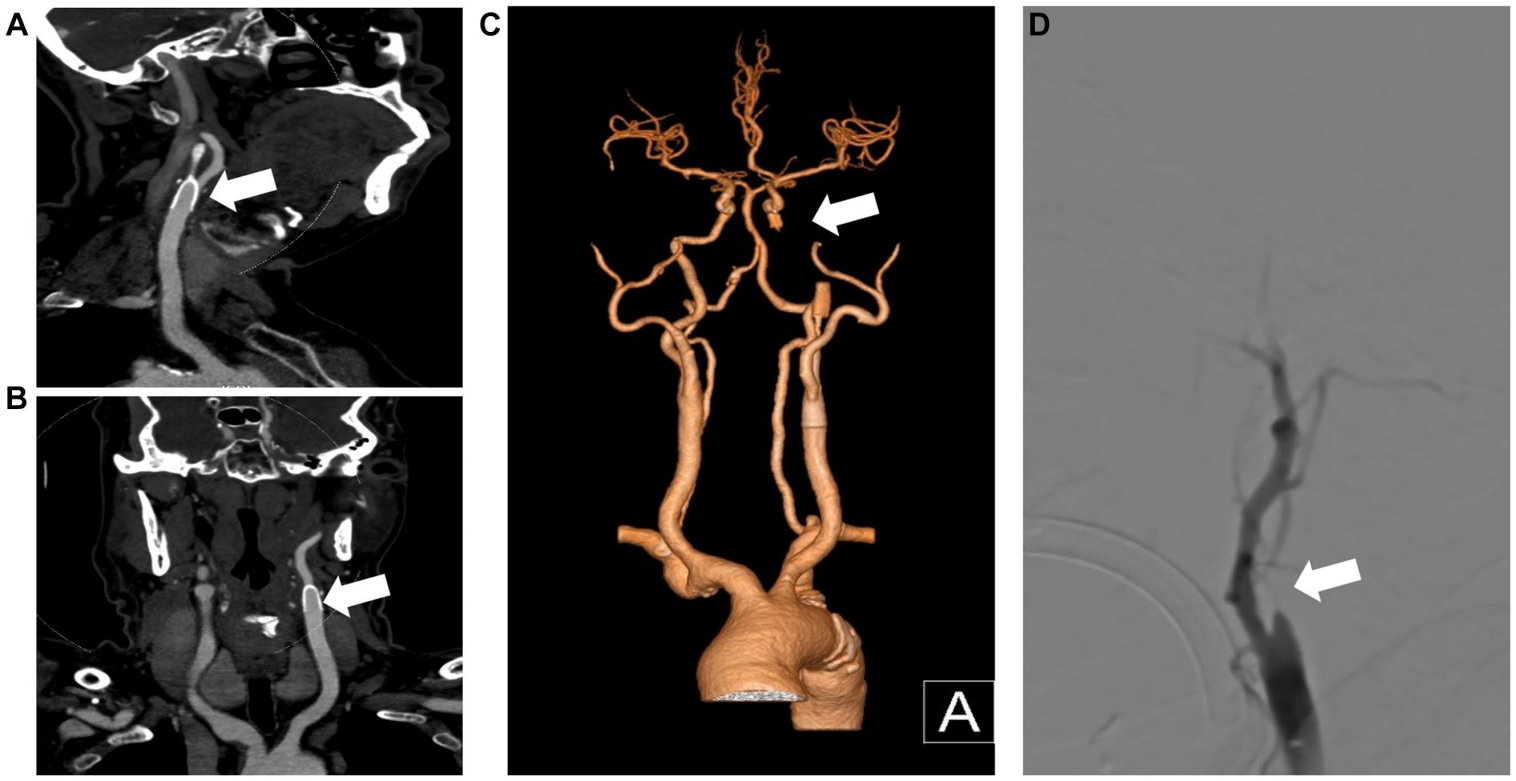
CTA and DSA show carotid stent thrombosis. The sagittal plane **(A)**, coronal plane **(B)** and 3D reconstruction **(C)** of the CTA show stent thrombosis. DSA confirmed left internal carotid artery thrombosis and stent occlusion **(D)**. CTA, computed tomography angiography; DSA, digital subtraction angiography; 3D, three-dimensional.

**Figure 4:**
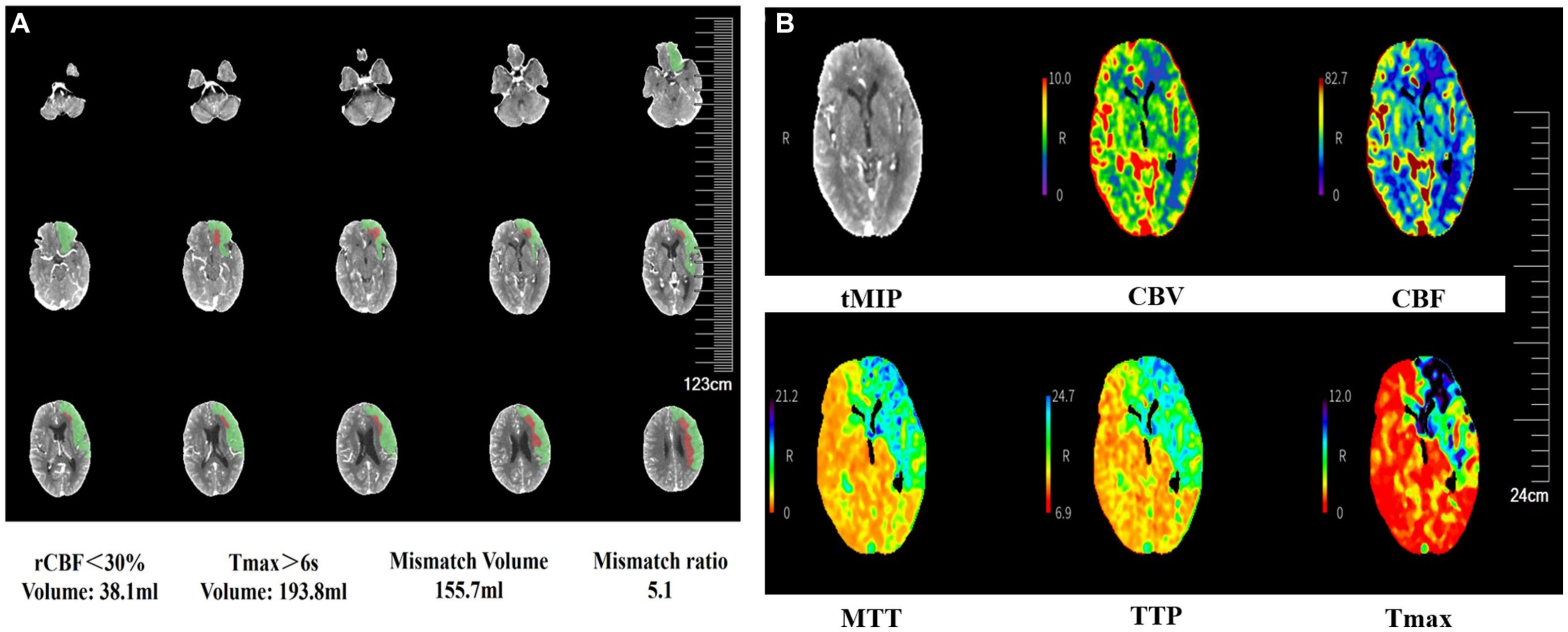
CTP images show cerebral blood flow following carotid stent thrombosis. CTP shows the ischemic penumbra after acute ischemia **(A)**. CBF and CBV decrease, and TTP and MTT increase in the left cerebral hemisphere, indicating left cerebral ischemia **(B)**. CTP, computed tomography perfusion; CBF, cerebral blood flow; CBV, cerebral blood volume; TTP, time-to-peak; MTT, mean transit time; rCBF, relative cerebral blood flow; Tmax, time to top; tMIP, temporal maximum intensity projection.

**Figure 5:**
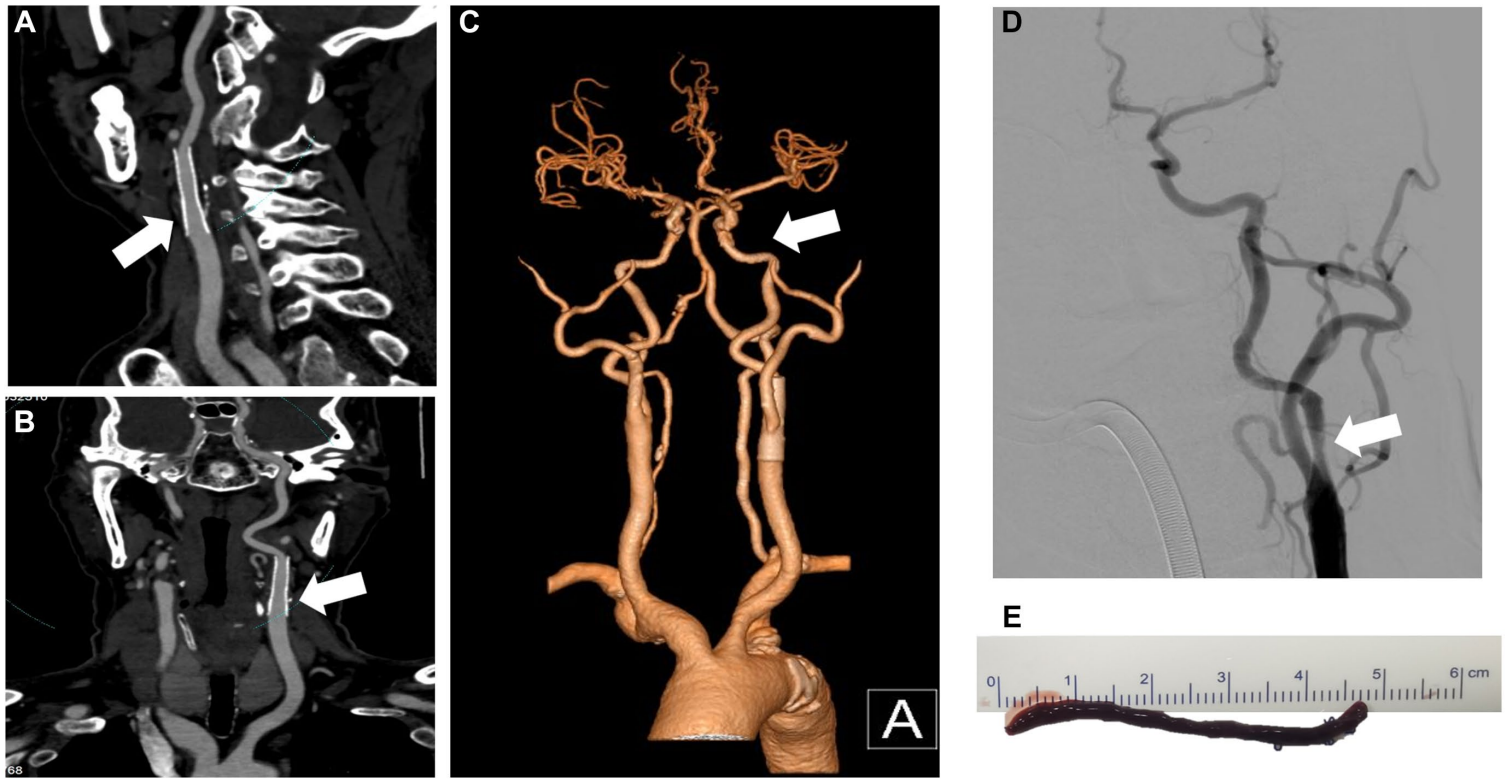
CTA and DSA show no residual stenosis and no thrombosis after mechanical thrombectomy. The sagittal plane **(A)**, coronal plane **(B)**, and 3D reconstruction **(C)** of the CTA show carotid artery recanalization. DSA shows no signs of thrombosis **(D)**. The 4.6-cm red blood clot was removed from the left internal carotid artery after mechanical thrombectomy **(E)**. CTA, computed tomography angiography; DSA, digital subtraction angiography; 3D, three-dimensional.

A 69-year-old man with hypertension and diabetes presented to our institution complaining of slurred speech and right limb weakness. The patient had a history of ischemic stroke with approximately 95% stenosis of his left ICA, and the carotid stent (XACT 7–9 mm × 40 mm; Boston Scientific, Natick, MA, United States) was placed at the site of the stenosis (Figure 2). Aspirin 100 mg and clopidogrel 75 mg once daily were given for only 7 days after CAS, and then he refused continuous dual antiplatelet therapy. After 1 day, he developed neurological deficits with hypersomnia, aphasia, and right hemiplegia. CTA and digital subtraction angiography (DSA) confirmed left ICA occlusion (Figure 3). In addition, computed tomography perfusion (CTP) showed that cerebral blood flow (CBF) and cerebral blood volume (CBV) decreased, and time-to-peak (TTP) and mean transit time (MTT) increased, which indicated the ischemia of the left cerebral hemisphere (Figure 4). The operation was then performed under general anesthesia. During the procedure, the 8-Fr guiding catheter was inserted into the left common carotid artery, and the occlusion was crossed with a Transend 0.014-inch microguidewire and a Rebar 18 microcatheter. Selective microcatheter angiography revealed that the stent occlusion was in the cervical segment of the ICA. A 4.0-mm Spider Fx CPD was positioned in the petrous segment of the ICA. The reperfusion catheter was then advanced into the stent to aspirate the thrombus. Lumpy thrombi were retrieved from the reperfusion catheter, and angiography showed complete recanalization within the stent, but residual stenosis within the stent was approximately 40%. Percutaneous transluminal angioplasty (PTA) was performed on the remaining stenosis in the stent using a 5 × 20 mm angioplasty balloon (14 atm, 3 s). Post-procedure angiography showed brisk flow through the internal carotid artery and cerebral circulation (Figure 5). His neurological symptoms were resolved at discharge with a NIHSS score of 4, and he made a full recovery and returned to normal daily life and work at a 3-month follow-up.

## Discussion

Cerebrovascular diseases have become one of the leading causes of death and disability worldwide with the aging of the population. Among these, ischemic stroke is the most common cause, accounting for over 70% of cases (7, 8). Previous studies showed that the incidence of ischemic stroke increased significantly in patients with carotid stenosis without surgical revascularization (2). The standard treatment regimen for carotid artery stenosis has not yet been established. Carotid endarterectomy is confirmed to be an effective and safe treatment regimen but carries periprocedural risks, including disabling stroke and death (9). Carotid artery stenting has emerged as an option for the treatment of carotid artery disease (10). Additionally, dual antiplatelet therapy and lipid-modulating agents are strongly recommended periprocedural (11).

According to the International Carotid Stenting Study (ICSS) clinical study, the long-term efficacy and safety were similar between stenting and endarterectomy for symptomatic carotid stenosis (12); however, the complications caused by CAS, such as stent thrombosis, plaque prolapse, residual stenosis, and incorrect stent deployment, have been reported. With the insertion of stents, the endangium was destroyed, followed by platelet aggregation and arterial thrombosis (13). In our institution, overall 262 (87.6%) patients with carotid artery stenosis received CAS between 2020 and 2022 according to symptoms, degree of stenosis, and risk factors, and the majority of patients were well tolerated, with approximately 5.3% experiencing severe complications, including stenting restenosis, hyperperfusion or hypoperfusion syndrome, and carotid stent thrombosis. No deaths occurred during the procedure. Of the 14 patients who suffered severe complications, six patients (2.3%) experienced CST, which was similar to previous reports of approximately 2.0% (14, 15).

CST has been identified as a rare but fatal complication after CAS, but there is currently no standard treatment for it. Although previous literature has demonstrated several potential treatment regimens, there has been no randomized clinical trial to evaluate the efficacy and safety of different treatment regimens. The key to treatment for CST is rapid vascular recanalization. Thrombolytics of streptokinase and tenecteplase were applied by Iancu et al. to treat CST and achieved favorable results; two CST patients had no neurological deficits or acute ischemic lesions at discharge (16). In 2000, the potent antiplatelet drug abciximab was first reported to be used for the resolution of in-stent thrombus (17). The combination of thrombolysis with alteplase and dethrombosis with abciximab was also confirmed to promote arterial recanalization and inhibit thrombosis recurrence (18). Anticoagulant therapy was also reported to treat CST. In 2013, Kanemaru et al. reported that a bladder cancer patient had a large in-stent thrombosis 6 days after CST and was then treated with antiplatelet agents and anticoagulant therapy with argatroban (60 mg/day) and warfarin, and in-stent thrombosis gradually shrank and disappeared 6 weeks after CAS (19). The main complication of either thrombolytic or anticoagulant therapy is hemorrhage. Endarterectomy and thrombosed stent removal ensure rapid restoration of cerebral blood flow and reduce the risk of recurrence; several cases of urgent stent removal with favorable results have been demonstrated in previous studies (20, 21); however, endarterectomy might lead to hyperperfusion syndrome and local hematoma. Mechanical thrombectomy is another potential therapy method (22). The difficulty of thrombectomy for CST is that the microguidewire needs to pass through the thrombus to avoid passing between the stent and the vascular wall. It is necessary to pay attention to the morphology of the head of the microguidewire during operation, and the relationship between the carotid stent and guidewire needs to be observed by multi-angle magnification fluoroscopy. During thrombus aspiration, we should focus on the association between the suction catheter and the stent to avoid serious stent displacement. In our participants, all six patients were symptomatic and had significant carotid stenosis degrees of more than 85% before CAS; however, with the treatment of stent implantation, the stenosis grade declined to 30–40%. Unfortunately, they developed CST within 6 days to 45 months following CAS, with a median NIHSS of 12 (range 8–25). All six patients were evaluated based on their last seen normal time and angiography and then underwent mechanical thrombectomy directly. With the treatment of thrombectomy, the neurological deficits improved significantly after the procedure, suggesting to us that it is effective to achieve revascularization with mechanical thrombectomy even with severe stenosis.

According to the previous literature, the potential pathophysiologic causes of CST include plaque protrusion, antiplatelet non-compliance, antiplatelet resistance, internal carotid artery dissection, iatrogenic injury, and hypercoagulable status. The stent type was also found to correlate with the formation of stent thrombosis. Of which, inadequate, discontinued, or resistant antiplatelet agents have been confirmed to be a significant reason (6, 13, 23). Clopidogrel and aspirin are the most frequently chosen dual antiplatelet therapy (DAPT) regimens for those who received neuroendovascular stent placement. Based on the most recent Society of NeuroInterventional Surgery (SNIS) Guidelines, clopidogrel is recommended for at least 3 months following CAS, and aspirin should be taken continuously (24). In our study, we recommend patients take double antiplatelet drugs (aspirin 100 mg/day and clopidogrel 75 mg/day) continually for at least 3 months after stenting. However, two (33.3%) patients stopped taking antiplatelet drugs within 9 days of stenting and three (50.0%) patients stopped taking aspirin arbitrarily 3 months later; the antiplatelet treatment interruption might be the most important reason for the occurrence of CST, suggesting that we should guide patients to receive normative antiplatelet treatment. Intensive medical management and a healthy lifestyle are important for stroke prevention and treatment (25, 26), and the randomized clinical trial of SPACE-2 indicated that adequate BMT was effective in asymptomatic carotid stenosis (11). In our study population, all patients had aggressive risk factors, including smoking (100%), hyperhomocysteinemia (100%), hypertension (66.7%), and hyperlipidemia (66.7%). During the treatment, six (100%) patients failed to quit smoking, and only one patient received standard antihypertensive therapy, which may increase the incidence of in-stent thrombosis. Therefore, it is important to encourage patients to follow standard medical treatment and maintain a healthy lifestyle to reduce the risk of CST. We propose that these patients with high-risk factors may benefit from the prolonged duration of the dual antiplatelet therapy and increasing the antiplatelet dose.

There were several limitations to our study. First, the study was retrospective and lacked control groups receiving other treatments, including CEA or BMT. Second, only six patients were included in our study, and the results need to be confirmed by larger samples. Third, we did not conduct long-term follow-up, which might lead to a lack of complications.

Overall, our study demonstrates that mechanical thrombectomy is an effective, feasible, and safe treatment regimen for patients with in-stent thrombosis after severe carotid artery stenosis, which has inspired us to validate these conclusions through longer follow-up and prospective studies with larger samples in the future.

## Data availability statement

The original contributions presented in the study are included in the article/supplementary material, further inquiries can be directed to the corresponding author.

## Ethics statement

The Ethics Review Committee of Xuzhou First People's Hospital. The studies were conducted in accordance with the local legislation and institutional requirements. Written informed consent for participation was not required from the participants or the participants' legal guardians/next of kin in accordance with the national legislation and institutional requirements. Written informed consent was obtained from the individual(s) for the publication of any potentially identifiable images or data included in this article.

## Author contributions

Y-MF: Data curation, Investigation, Methodology, Writing – review & editing, Formal analysis, Software. H-YL: Data curation, Funding acquisition, Investigation, Methodology, Writing – review & editing. Y-YX: Conceptualization, Project administration, Resources, Supervision, Validation, Writing – original draft.
